# HEART Pathway Accelerated Diagnostic Protocol Implementation: Prospective Pre-Post Interrupted Time Series Design and Methods

**DOI:** 10.2196/resprot.4802

**Published:** 2016-01-22

**Authors:** Simon A Mahler, Gregory L Burke, Pamela W Duncan, Larry D Case, David M Herrington, Robert F Riley, Brian J Wells, Brian C Hiestand, Chadwick D Miller

**Affiliations:** ^1^Wake Forest School of MedicineDepartment of Emergency MedicineWinston Salem, NCUnited States; ^2^Wake Forest School of MedicinePublic Health SciencesWinston Salem, NCUnited States; ^3^Wake Forest School of MedicineDepartment of Neurology, Sticht Center on Aging, Gerontology, and Geriatric MedicineWinston Salem, NCUnited States; ^4^Wake Forest School of MedicineDepartment of Internal Medicine, Division of Cardiovascular MedicineWinston Salem, NCUnited States; ^5^Wake Forest School of MedicineTranslational Science Institute, Family and Community Medicine, Epidemiology and PreventionWinston Salem, NCUnited States

**Keywords:** chest pain, decision support technique, interrupted time series analysis, acute coronary syndrome, implementation methods, electronic medical records

## Abstract

**Background:**

Most patients presenting to US Emergency Departments (ED) with chest pain are hospitalized for comprehensive testing. These evaluations cost the US health system >$10 billion annually, but have a diagnostic yield for acute coronary syndrome (ACS) of <10%. The history/ECG/age/risk factors/troponin (HEART) Pathway is an accelerated diagnostic protocol (ADP), designed to improve care for patients with acute chest pain by identifying patients for early ED discharge. Prior efficacy studies demonstrate that the HEART Pathway safely reduces cardiac testing, while maintaining an acceptably low adverse event rate.

**Objective:**

The purpose of this study is to determine the effectiveness of HEART Pathway ADP implementation within a health system.

**Methods:**

This controlled before-after study will accrue adult patients with acute chest pain, but without ST-segment elevation myocardial infarction on electrocardiogram for two years and is expected to include approximately 10,000 patients. Outcomes measures include hospitalization rate, objective cardiac testing rates (stress testing and angiography), length of stay, and rates of recurrent cardiac care for participants.

**Results:**

In pilot data, the HEART Pathway decreased hospitalizations by 21%, decreased hospital length (median of 12 hour reduction), without increasing adverse events or recurrent care. At the writing of this paper, data has been collected on >5000 patient encounters. The HEART Pathway has been fully integrated into health system electronic medical records, providing real-time decision support to our providers.

**Conclusions:**

We hypothesize that the HEART Pathway will safely reduce healthcare utilization. This study could provide a model for delivering high-value care to the 8-10 million US ED patients with acute chest pain each year.

**ClinicalTrial:**

Clinicaltrials.gov NCT02056964; https://clinicaltrials.gov/ct2/show/NCT02056964 (Archived by WebCite at http://www.webcitation.org/6ccajsgyu)

## Introduction

### Background

Current care patterns for acute chest pain fail to focus health system resources, such as hospitalization and cardiac testing, on patients most likely to benefit. Each year, approximately 8-10 million patients complaining of chest pain present to an Emergency Department (ED) in the United States [1]. To avoid missing acute coronary syndrome (ACS), ED providers liberally hospitalize patients with acute chest pain for comprehensive cardiac evaluations (serial cardiac biomarkers and stress testing or angiography). However, <10% of these patients are ultimately diagnosed with ACS [2-6], and this pervasive overtesting costs an estimated US $10-13 billion annually [5,7]. Current guidelines for the management of suspected ACS recommend provocative or anatomic testing as a default strategy, serving to reinforce this overtesting behavior [7].

The Chronic Care Model, as adopted from Wagner’s work, identifies the use of evidence-based clinical decision support (CDS) systems as a way to address health system needs and improve health care delivery in chronic conditions such as cardiovascular disease [8-11]. Consistent with this model, accurate ACS risk stratification care pathways are designed to eliminate unnecessary testing and improve quality of care by decreasing false-positive results, nondiagnostic testing, exposure to radiation, and excess costs [12]. The history/ECG/age/risk factors/troponin (HEART) Pathway is an accelerated diagnostic protocol (ADP), which combines a clinical decision aid (the HEART score; Table 1) [13-15], with 2 serial troponin measurements, to identify patients with chest pain who can safely be discharged without objective cardiac testing (stress testing or angiography), either urgently or during follow-up care. Prior studies have established that use of the HEART Pathway reduces cardiac testing by >20%, while maintaining an acceptably low adverse event rate [15-17]. What is needed now is a rigorous evaluation of the implementation of the HEART Pathway into *real-world* clinical settings to determine its effectiveness.

**Table 1:** The HEART score.

Category	Description	Points
History	Highly suspicious	2
	Moderately suspicious	1
	Slightly suspicious	0
ECG	Significant ST-depression	2
	Nonspecific repolarization abnormality	1
	Normal	0
Age	>65	2
	45-65	1
	<45	0
Risk Factors	3 or more risk factors	2
	1-2 risk factors	1
	No risk factors	0
Troponin	>3x normal limit	2
	1-3x normal limit	1
	<normal limit	0
Total		

### Objectives and Hypotheses

In this paper, we describe the rationale and methods utilized to test the effectiveness of the HEART Pathway ADP within a health system consisting of three diverse hospital settings. We hypothesize that implementation of the HEART Pathway will significantly reduce hospitalizations and the rate of objective cardiac testing among low-risk patients, without increasing adverse cardiac events.

## Methods

### Study Design

This prospective pre-post interrupted time series design compares the risk stratification of patients with acute chest pain before and after implementation of the HEART Pathway ADP. Wake Forest Baptist Health is a three-hospital academic health system located in the Piedmont Region of North Carolina. It consists of a large quaternary academic medical center with approximately 104,000 ED visits annually, a small community hospital with an annual ED volume of about 37,000 patients, and a free standing ED in an adjacent rural county with approximately 12,000 annual ED visits. This study is approved by the Internal Review Board of the sponsoring organization and is registered with clinicaltrials.gov (NCT02056964).

### Eligibility

The target population is adult patients with acute chest pain, in which the provider is concerned about possible ACS, but does not have evidence of an ST-segment elevation myocardial infarction (STEMI) on electrocardiogram (ECG). Therefore, adult patients (aged ≥ 21 years) with acute chest pain, provider-ordered troponins, and without STEMI will be included. Based on STEMI rates at Wake Forest Baptist Health, we expect <5% of patients with acute chest pain to be excluded due to ECG criteria.

### Study Timeline

**Figure 1:**
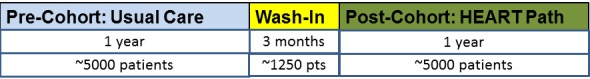
HEART Pathway Implementation prospective cohort design.

Pre- and post-HEART Pathway ADP implementation cohorts will each accrue patients with acute chest pain for 1 year, with a 3-month wash-in period (Figure 1). Data will be collected electronically from all patients using health system electronic medical records (EMRs), and claims data from insurers will be used on a subset of patients insured by Blue Cross Blue Shield (BCBS) of North Carolina (the largest insurer in the state), MedCost, or North Carolina Medicaid.

### HEART Pathway Implementation

**Figure 2:**
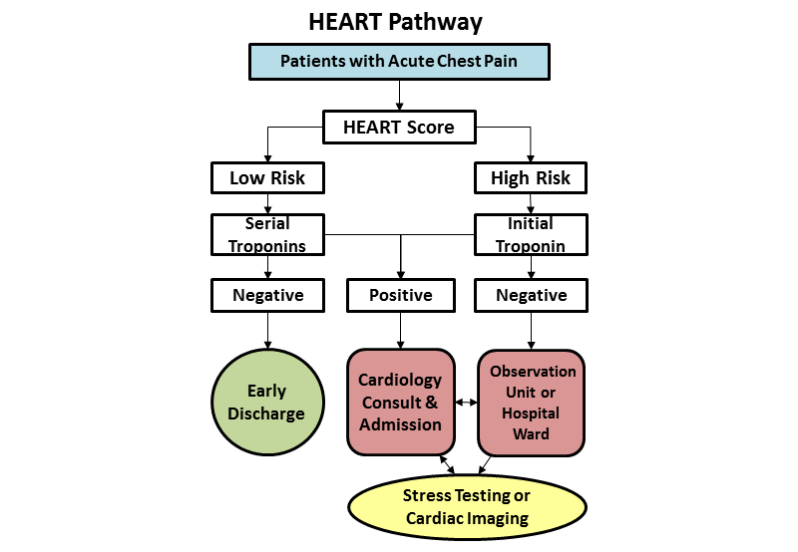
HEART Pathway algorithm.

The HEART Pathway intervention will incorporate elements of the Chronic Care Model framework (decision support and clinical information systems) by providing test ordering and disposition decision support to ED practitioners and personalized care planning for patients with acute chest pain [8,18,19]. This intervention uses a clinical decision aid (the HEART score; Table 1) [13-15], with 2 serial troponin measurements obtained at 0 and 3 hours after ED presentation. The HEART Pathway algorithm (Figure 2) will be integrated into the EMR system, EPIC (Madison, WI, USA), as an interactive CDS tool. A model window (on-screen pop-up) will display the HEART Pathway tool as a Best Practice Advisory when clinically indicated. The child window will require interaction before the user may return to the parent window in the EMR, but will not force the user to utilize the HEART Pathway tool (the alert will be presented as a *soft-stop*). The modal window will appear when a provider has ordered a troponin test on a patient with a chief complaint of “chest pain” or “heart problem.” The on-screen pop-up will facilitate use of the HEART Pathway CDS tool, by leveraging the normal work-flow of ED providers. For patients presenting with other symptoms that are concerning for ACS (ie, dyspnea, left arm pain, or jaw pain), providers will be encouraged to access the HEART Pathway tool manually. The HEART Pathway tool will be accessible from the EMR’s main menu (the ED Navigator).

Once opened, by pop-up or manually, the HEART Pathway tool will prompt providers to answer a series of questions to determine eligibility and calculate a HEART score on eligible patients. The software will calculate a HEART score based on provider responses and give recommendations on further care based on the HEART Pathway (Figure 2). Patients with a low-risk HEART score (3 or less) and negative troponins at 0 and 3 hours will be identified as a population not requiring objective cardiac testing who can be discharged safely from the ED. Patients deemed at high-risk by the HEART Pathway or with a positive troponin (>99th percentile) will be identified for further testing and/or admission. HEART Pathway use will be tracked using weekly EMR reports. This report identifies all patients who meet inclusion criteria, determines whether the pop-up was activated, and whether the HEART Pathway tool was completed by the ED provider. Noncompliant providers, those that ignore the HEART Pathway pop-up, or those that fail to complete the HEART Pathway decision support tool assessment will receive notification and corrective education via email.

### Data Collection

**Figure 3:**
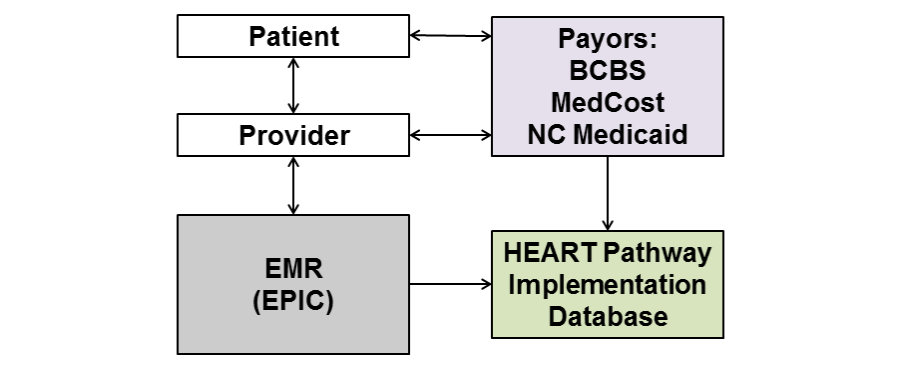
Flow of data into the HEART Pathway Implementation Database.

The effectiveness of the HEART Pathway will be assessed using electronic outcome surveillance. EMR data will be collected on all patients with chest pain. Insurance claims data will be collected on patients with Medicaid, MedCost, and BCBS of North Carolina. Data will include patient demographics, past medical history, cardiovascular risk factors, ECG results, troponin results, HEART score and HEART Pathway assessments, ED and discharge diagnoses, disposition, index visit length of stay, objective cardiac testing (stress testing or angiography) at the index visit or within 30 days, recurrent ED visits, readmissions, and myocardial infarction (MI) or death within the follow-up period. Data extraction from the EMR data warehouse (Clarity) will be automated and programmed to pull prespecified data points into our database on patients meeting eligibility criteria on a weekly or monthly basis (Figure 3). Electronic surveillance leverages our informatics strengths and data sharing relationships with insurers, to provide efficient and accurate outcome data. Data accuracy is enhanced by avoiding recall bias inherent in other follow-up methods such as telephone calls. Electronic surveillance also improves feasibility by allowing programmable follow-up on a large number of patients at a low cost. Inclusion of Medicaid patient claims should ensure that our data surveillance includes economically disadvantaged patients and is generalizable to the patient populations of other hospitals. Any discrepancies between sources of data will be adjudicated by study investigators blinded to patient pre- or post-intervention cohort participation. Based on prior studies, we expect 80% of patients to have follow-up data from the EMR or insurer claims. The Social Security Death Master File will be used to search for participants without follow-up data.

The preintervention cohort will be identified using the same inclusion criteria (aged ≥ 21 years with a complaint of chest pain and a troponin ordered without STEMI on ECG) for 1 year prior to integration of the HEART Pathway into cardiovascular care delivery at Wake Forest Baptist Health. The same data elements described above will be abstracted from the EMR and claims databases from BCBS, MedCost, and Medicaid.

### Outcome Measures

Pre- and post-HEART Pathway cohorts will be compared for safety and health care utilization outcomes. The primary outcome will be hospitalization rate at 30 days for patients presenting with acute chest pain. Secondary outcomes will include index hospitalization rate, index and 30-day objective cardiac testing, hospital length of stay (LOS), recurrent ED visits, and nonindex admissions for chest pain. Outcomes will be assessed during the index visit and for 30 days thereafter.

Hospitalization will be defined as an inpatient admission or observation stay. Objective cardiac testing will be defined by any stress testing modality, coronary computed tomography angiography, or invasive coronary angiography. The modalities routinely available at Wake Forest Baptist Health include exercise ECG, exercise stress echocardiogram, dobutamine stress echocardiogram, coronary computed tomography angiography, stress nuclear imaging, stress cardiac magnetic resonance imaging, or invasive coronary angiography. LOS will be the time from ED arrival to hospital discharge for all patients, whether admitted or not. A recurrent visit to the ED will be defined as any patient revisiting the ED with chest pain or other symptoms suggestive of ACS within the 30-day follow-up period.

Safety outcomes will include death and acute MI within 30 days. Missed adverse events will be defined as death or MI occurring in patients discharged from the ED without objective cardiac testing. The definition of MI will be based on the Third Universal Definition of MI, which includes a rising or falling pattern of troponin with a cutoff representing the 99th percentile reference value with a coefficient of variation <10% [20].

### Statistical Analyses

Logistic regression models, which include potential patient-level confounders such as age, sex, race, ethnicity, insurance status, and ACS risk factors in addition to the intervention indicator, will be used to assess the effect of the HEART Pathway intervention on hospitalizations (and other dichotomous outcomes such as objective cardiac testing and recurrent ED visits). As secular trends represent a threat to internal validity (observed differences between groups could be from time trends rather than the intervention), we will include the time since the start of the study in the models, as well as the time by intervention interaction. This will allow us to assess the effect of the HEART Pathway implementation on the proportion of patients hospitalized, as well as its effect on the time trend after implementation.

Continuous outcomes will be analyzed using multiple linear regression models including potential confounders, time, the intervention indicator (pre- or post-HEART Pathway implementation), and the time by intervention interaction as described above. LOS tends to be skewed, so we will use some transformation (log, rank, etc) in the analyses if necessary. Residuals will be examined to ensure the model assumptions (linearity, homoscedasticity, and normality) are met. Not all patients will have electronic follow-up data. The default assumption will be that patients without follow-up data from the EMR, insurers, or Social Security Death Masterfile did not suffer adverse events. Sensitivity analyses will be used to assess the impact of missing data on our results. The analyses will be repeated assuming all patients with missing follow-up data had follow-up events (hospitalization, cardiac testing, etc). Multiple imputation will be used to conduct sensitivity analyses that generate complete datasets under a variety of assumptions regarding the rates of outcomes in the two periods. All analyses will be performed using SAS 9.4 (Cary, NC). *P*<0.05 will be considered significant.

### Sample Size

A sample size of approximately 10,000 patients, (5000) pre- and (5000) post-intervention, is anticipated. Based on prior studies it is expected that 80% (4000 patients/group) will have follow-up data from the EMR or insurer claims. With this sample size we will be able to estimate safety event rates for each study period to ±0.33% with 95% confidence (assuming an event rate of 1%). Based on our prior studies, we expect a hospitalization rate of 53% and an objective cardiac testing rate of 83% among hospitalized patients during the preintervention period [16,21]. This study will be adequately powered to detect reductions in hospitalization and objective cardiac testing rates of <5%, with 90% power at the 5% two-sided level of significance. We anticipate larger effects [16,17,22].

### Multidisciplinary Collaboration

To facilitate implementation, we have created the Wake Forest HEART Pathway Integration Team consisting of key stakeholders within health system leadership, medical school leadership, and across the disciplines of public health, medical informatics, cardiology, primary care, nursing, and emergency medicine. Through this collaborative effort we will not only engage key stakeholders in the integration of the HEART Pathway with education and care delivery at Wake Forest Baptist Health, but also gain great insights into the potential barriers and facilitators for widespread dissemination and implementation of similar evidenced-based health system quality improvement initiatives across US medical centers.

## Results

### Preliminary Data

To evaluate the HEART Pathway prior to implementation, we analyzed registry data from 1070 low-risk chest pain patients in an ED-based Observation Unit at Wake Forest Baptist Health. The HEART Pathway identified all 12 patients with major adverse cardiac events at 30 days (100% sensitivity, 95% CI 72-100%) and could have identified 879 of 1070 patients (82%, 95% CI 80-84%) for early discharge without objective testing [16]. Next, we analyzed data from the Myeloperoxidase In the Diagnosis of Acute Coronary Syndromes (MIDAS) Study [23], a multicenter cohort which included 991 participants with suspected ACS from 18 US EDs and data for HEART Pathway risk assessment. ACS was present in 220 of 991 patients (22% of the cohort). In this cohort, the HEART Pathway identified 218 of 220 patients (99% sensitivity, 95% CI 97-100%) with ACS (cardiac death, MI, or unstable angina) within 30 days and identified 200 of 991 patients (20%, 95% CI 18-23%) for early discharge [17]. A lower early discharge rate in MIDAS compared with our first study is explained by the higher prevalence of ACS events in the MIDAS cohort (22% vs 1%). Most importantly, the HEART Pathway had 99% sensitivity in this higher-risk population, suggesting it can be applied broadly to all patients undergoing ACS evaluation. Finally, we conducted the HEART Pathway Randomized Controlled Trial in which 282 adult patients with acute chest pain were randomized to risk stratification via the HEART Pathway ADP or usual care, based on American College of Cardiology/American Heart Association guidelines. In this trial, use of the HEART Pathway decreased objective cardiac testing at 30 days, reduced median LOS by 12 hours (9.9 vs 21.9 hours, *P*<.01), and increased early discharges. In the usual care group 97 of 141 patients had objective cardiac testing compared with the HEART Pathway group, which had testing in 80 of 141 patients (a difference of 12.1%, 68.8% vs 56.7%, *P*=.048). In the usual care group, 26 of 141 patients had an early discharge, while 56 of 141 had early discharge in the HEART Pathway group (a difference of 21.3%, 39.7% vs 18.4%, *P*<.001). No patients identified by the HEART Pathway for early discharge had adverse cardiac events within 30 days and the HEART Pathway was not associated with increased recurrent care [22].

### HEART Pathway Implementation

At the writing of this paper, data for this implementation study have been collected on >5000 patient encounters. The HEART Pathway has been fully integrated into health system EMRs, providing real-time decision support to our providers. Data infrastructure has been built such that patients meeting inclusion criteria are included in a registry, including 30-day electronic-surveillance data, which will be integrated with insurer claims data.

## Discussion

### Study Rationale

This paper describes the design of a prospective cohort study which will determine the effectiveness of the HEART Pathway ADP in safely reducing health care utilization (hospitalizations, objective cardiac testing, hospital length of stay, etc) among ED patients with acute chest pain. This study is timely given the high cost of delivering care to patients with acute chest pain and the current focus on delivering high-value care. The National Quality Strategy, outlined in the Affordable Care Act, focuses on increasing health care quality while simultaneously lowering costs [24]. To adapt to this changing health care landscape, health systems must develop effective methods of translating efficient evidence-based protocols, such as the HEART Pathway, into clinical practice [25-27].

Implementation of the HEART Pathway could improve the value of care for patients with chest pain by decreasing unnecessary health care utilization, false-positive/nondiagnostic testing, radiation, and costs [12]. Our prior analyses of the HEART Pathway, in combination with the HEART Score validation studies [14,15], provide efficacy data on over 7000 patients, and suggest that the HEART Pathway can have a large impact on avoiding testing in low-risk patients, yet retains high sensitivity when applied to higher-risk patients. Furthermore, the HEART Pathway’s ability to rapidly identify patients for early discharge and its overall ease of use make adoption in an ED setting feasible. Our experience with the HEART Pathway suggests that patients identified for early discharge will have significant reductions in index LOS and cost [22]. What is needed now is a prospective cohort study, as described in this paper, which will determine the effectiveness of the HEART Pathway by prospectively implementing it in a *real-world* clinical setting.

Nontraditional aspects of this study design include its pre-post interrupted time series design, multidisciplinary team implementation, CDS integrated into the EMR, automated prospective data capture, and electronic surveillance of outcomes (including partnering with insurers for claims data). These features will allow the passive accrual and surveillance of over 10,000 patients. Furthermore, this design will provide a template for others interested in pragmatic testing of care pathways within health systems.

### Limitations

The design of this study has some limitations when compared to a traditional randomized design. Secular trends and provider maturation effects are potential threats to the validity of our pre-post time series design. The electronic surveillance used in this study may increase loss-to-follow-up rates compared with traditional methods of follow-up. However, to determine effectiveness, the HEART Pathway must be utilized in an *all-comers* ED patient population. Randomized clinical trials, due to the consent process, have an inherent selection bias which can threaten the validity of an effectiveness study. Furthermore, given the size and scope of this implementation study, this design is more feasible and cost effective than a clinical trial.

### Conclusions

We hypothesize that this study will demonstrate that HEART Pathway implementation within a health system will result in meaningful reductions in health care utilization without compromising patient safety. We expect the HEART Pathway to decrease hospitalizations for comprehensive cardiac evaluations and the rate of objective cardiac testing among patients with a low pretest probability for ACS. In addition, our prior data suggest that the HEART Pathway will shorten hospital LOS for patients with acute chest pain, which should translate into significant cost savings and efficiency gains. Success of this study could provide a model for health systems to provide high-value care to 8-10 million patients who present to US EDs with acute chest pain each year.

## Multimedia Appendix 1

Peer Review from AAMC/Donaghue Foundation Capacity-Building Grant Opportunity for Academic Medical Centers.

## Abbreviations

ACS: acute coronary syndrome
ADP: accelerated diagnostic protocol
BCBS: Blue Cross Blue Shield
CDS: clinical decision support
ECG: electrocardiogram
ED: Emergency Department
EMR: electronic medical record
HEART: history/ECG/age/risk factors/troponin
LOS: length of stay
MI: myocardial infarction
MIDAS: Myeloperoxidase In the Diagnosis of Acute Coronary Syndromes
STEMI: ST-segment elevation myocardial infarction
