# Supplementary material for: HEART Pathway Accelerated Diagnostic Protocol Implementation: Prospective Pre-Post Interrupted Time Series Design and Methods
Source: JMIR Res Protoc. 2016 Jan 22;5(1):e10. doi: 10.2196/resprot.4802 (PMC4744329; doi:10.2196/resprot.4802)
Supplement: Supplementary file 1 [file resprot_v5i1e10_app1.pdf]

AAMC

Association of American Medical Colleges
2450 N Street, N.W., Washington, D.C. 20037-1127
T 202 828 0400 **F** 202 828 1125
www.aamc.org

August 2, 2013

Simon A. Mahler, MD
Medical Center Boulevard
Receiving-Commons Bldg
Wake Forest Univ Health Services
Winston-Salem, NC 27157

Dear Dr. Mahler,

We are pleased to inform you that The Heart PATHWAY: Bridging the Gap between Operations, Research and Education has been selected for funding through *Advancing Effectiveness Research and Implementation Science in Our Own Backyards: AAMC/Donaghue Capacity-Building Grant Opportunity for Academic Medical Centers*.

As noted in the RFP, fund distribution is contingent upon a signed contract with the AAMC and documentation of current IRB approval of the project. We will send the contract to you under separate cover. I will also contact you to arrange a discussion to this end, and to organize a meeting around the AAMC evaluation of the project.

Our scientific peer review committee faced a difficult task in selecting two proposals from among the many interesting proposals submitted. Their enthusiasm for your proposal is captured in the review committee comments which are attached below. We look forward to working with you in the years ahead to shepherd this outstanding project to completion.

Sincerely,

Alexander K. Ommaya, D.Sc.
Senior Director, Clinical Effectiveness and Implementation Research

Review Committee Comments

Project Title: The HEART Pathway: Bridging the Gap between Operations, Research and Education

PI: Simon Mahler, MD, MS; Assistant Professor, Department of Emergency Medicine; Wake Forest University Health Services

Qualifications of the leadership and investigative team

Dr. Simon A. Mahler is well qualified to head this program. He is an established scientist in the area of cardiac assessment in the ED and has published several articles. He has research support from AHA and NIH on cardiac assessment. Dr. C. Miller, the co-investigator is also the director of clinical research / ED. Thus, the team is well experienced with a track record and departmental success.

Rigor of study design and analytic plan

The weak pre-post design is well justified and need for it documented. This is a carefully designed implementation study and the analytic plan is appropriate and straightforward. Power estimates are satisfactory. Will look at primary outcomes in 30 days for cardiac events, secondary outcomes analysis of utilization of cardiac testing, LOS, ED visits. Hospitalizations will be reviewed for both inpatient and ovservational status. Strong evidence base for intervention. Some of death data not specified (at home, out of area).

Likelihood that approach will be innovative and outcomes will have impact

This is not a highly innovative approach, the Heart Pathway has been around for some time. What is significant is the incredible institutional support to train/educate and support this program. The impact at Wake Forest will be significant and long lasting if the outcomes data supports the program. The project could be a model for organizational commitment to solving an important clinical problem that controls cost and provides acceptable safe care. The ideas behind it are solid and the premise that we should not be over-testing low-risk patents is compelling.

Inclusion of education and training elements

The program is driven by well informed team members. There is heightened awareness of this Heart Pathway implementation by training all team members, residents, nurses, physicians, etc.

Commitment and engagement of health system partner

Dr. Mahler has strong support of Wake Forest/Baptist Medical Center as evidenced by the lettes from the CEO/COO/Dean/CIO. With various third party insurers, i.e., Medcost, BlueCross BlueShield.

It appears that the team is well integrated with the health system and has already obtained the appropriate buy-in. It should be verified before funding that the IRB will, indeed, waive informed consent for this sturdy, because it is research and not just quality improvement.

Additional review comments

The program will be most successful where there are well run Emergency Departments that are supported by an observation program where the assignments can be completed. The impact score is limited because the expectation of < 1% missed diagnoses. Suggest DSMB. Low miss rate for HEART vs. usual care.
